# Cognitive, emotional and somatic symptoms among patients with chronic posttraumatic headache. A controlled study

**DOI:** 10.1186/1129-2377-14-S1-P155

**Published:** 2013-02-21

**Authors:** D Kjeldgaard, H Forchhammer, R Jensen

**Affiliations:** 1 Danish Headache Centre, Denmark; 2 Glostrup University Hospital, Denmark

## Introduction and aim

Patients with chronic posttraumatic headache (CPTH) after mild traumatic brain injury may develop multiple emotional, cognitive and somatic symptoms but these symptoms are often unspecific and reported among other headache patients, too. The aim is to investigate whether CPTH patients report more symptoms in general and with a higher severity of these symptoms than other headache patients.

## Method

The Rivermead Post Concussion Symptoms Questionnaire (RPQ) by King et. al.[1], is a validated instrument, which measures the severity and number of cognitive, emotional and somatic symptoms commonly experienced after head injury. The 16 symptoms are rated on a 5-point Likert scale (0=not experienced at all, 1=no more of a problem, 2=a mild problem, 3=a moderate problem, 4=a severe problem). All RPQ items were summed to a total score. The symptoms were divided into the three sub-scales: cognitive, emotional and somatic.

## Results

78 CPTH patients and 45 age- and sex matched patients with other headaches (control group) from the Danish Headache Centre were included. The F/M ratio was 6/4in both groups, the mean age was 34 and 38 years resp, and headache frequency 26 vs. 24 days/month resp. The CPTH group, compared to the control group, showed a significantly higher number of symptoms and severity of the symptoms on all the three sub-scales (severity 0-4 scale): cognitive (2.5 vs. 1.5), emotional (1.7 vs. 1.2) and somatic (1.9 vs. 1.5).

## Discussion

These results (being part of a larger study) suggest that CPTH patients compared to other headache patients with equally number of headache days are significantly more burdened by cognitive, emotional and somatic symptoms, and present a greater severity and impact on their life compared to other headache patients. This indicates that treatment of CPTH should focus broader than just headache treatment, indicating the need for multidisciplinary treatment.
